# Capillary Transit Time Heterogeneity Is Associated with Modified Rankin Scale Score at Discharge in Patients with Bilateral High Grade Internal Carotid Artery Stenosis

**DOI:** 10.1371/journal.pone.0158148

**Published:** 2016-06-23

**Authors:** Sibu Mundiyanapurath, Peter Arthur Ringleb, Sascha Diatschuk, Mikkel Bo Hansen, Kim Mouridsen, Leif Østergaard, Wolfgang Wick, Martin Bendszus, Alexander Radbruch

**Affiliations:** 1 Department of Neurology, University Hospital Heidelberg, Heidelberg, Germany; 2 German Cancer Research Center, Department of Radiology, Heidelberg, Germany; 3 Center of Functionally Integrative Neuroscience and MINDLab, Institute of Clinical Medicine, Aarhus University, Aarhus, Denmark; 4 Department of Neuroradiology, Aarhus Univesity Hospital, Aarhus, Denmark; 5 CCU Neurooncology, German Cancer Consortium (DKTK) & German Cancer Research Center (DKFZ), Heidelberg, Germany; 6 Department of Neuroradiology, University Hospital Heidelberg, Heidelberg, Germany; Chinese Academy of Sciences, CHINA

## Abstract

**Background and Purpose:**

Perfusion weighted imaging (PWI) is inherently unreliable in patients with severe perfusion abnormalities. We compared the diagnostic accuracy of a novel index of microvascular flow-patterns, so-called capillary transit time heterogeneity (CTH) to that of the commonly used delay parameter T_max_ in patients with bilateral high grade internal carotid artery stenosis (ICAS).

**Methods:**

Consecutive patients with bilateral ICAS ≥ 70%^NASCET^ who underwent PWI were retrospectively examined. Maps of CTH and T_max_ were analyzed with a volumetric approach using several thresholds. Predictors of favorable outcome (modified Rankin scale at discharge 0–2) were identified using univariate and receiver operating characteristic (ROC) curve analysis.

**Results:**

Eighteen patients were included. CTH ≥ 30s differentiated best between patients with favorable and unfavorable outcome when both hemispheres were taken into account (sensitivity 83%, specificity 73%, area under the curve [AUC] 0.833 [confidence interval (CI) 0.635; 1.000]; p = 0.027). The best discrimination using T_max_ was achieved with a threshold of ≥ 4s (sensitivity 83%, specificity 64%, AUC 0.803 [CI 0.585;1.000]; p = 0.044). The highest AUC was found for left sided volume with CTH ≥ 15s (sensitivity 83%, specificity 91%, AUC 0.924 [CI 0.791;1.000]; p = 0.005).

**Conclusion:**

The study suggests that CTH is superior to T_max_ in discriminating ICAS patients with favorable from non-favorable outcome. This finding may reflect the simultaneous involvement of large vessels and microvessels in ICAS and underscore the need to diagnose and manage both aspects of the disease.

## Introduction

In ischemic stroke, brain tissue is damaged by hypoxia resulting from severe hypoperfusion. This perfusion deficit has been studied using perfusion weighted imaging (PWI) for many years [1,2]. PWI can be used for the prediction of outcome [3,4], as well as for the selection of patients for acute recanalization procedures [5]. Measurement of cerebral blood flow (CBF), cerebral blood volume, mean transit time (MTT) and time-to-peak have been used to characterize perfusion in stroke [2], and more recently, the time to the maximum of the residue curve (T_max_) has been introduced as a surrogate of hypoperfused tissue [6,7]. To determine Tmax, the tissue residue curve must first be calculated by deconvolution of the tissue concentration time curve in each voxel and a global arterial input function (AIF) using singular value decomposition (SVD) [8]. SVD methods include standard deconvolution (sSVD), oscillation index SVD (oSVD) and circulant SVD (cSVD). The latter two are performed with block-circulant matrix for deconvolution with and without minimizing oscillations of the residue function, respectively [9]. Recently, the analysis of PWI data was extended to include capillary transit time heterogeneity (CTH) in a flexible, model-based bayesian framework, which has proved robust across realistic signal to noise ratios [10,11]. The CTH parameter provides information of the distribution of capillary transit times relative to their mean MTT within each voxel. Whereas MTT is an estimate of net tissue perfusion, CTH affects the extraction efficacy of oxygen from blood, in the way that high CTH leads to functional shunting of oxygenated blood through the vasculature [12]. It has previously been shown that capillary flow patterns are disturbed in acute ischemic stroke [13] and this phenomenon was recently hypothesized to play an important role in cerebral ischemia–reperfusion injury [14]. Indeed a recent study suggests that CTH must be known to account for the oxygen extraction fraction (OEF) as measured by positron emission tomography (PET) in ICAS patients [15].

Internal carotid artery stenosis (ICAS) is a frequent cause of ischemic stroke. Using PWI in patients with ICAS can be challenging, as the AIF will be distorted due to delay and dispersion of the bolus arrival. cSVD and oSVD seem to be beneficial in this situation as they are less delay sensitive [9]. However, other authors state that there is no relevant difference between the use of sSVD and oSVD [6]. While unnormalized TTP was reported to show clinically irrelevant increases in patients with ICAS [16], we found that T_max_ and normalized TTP depict clinically relevant hypoperfusion [17]. Nonetheless, it is currently unknown if these results can be applied to bilateral high grade ICAS, which often results in severe hypoperfusion and decreased cerebrovascular reactivity [18].

The aim of the current study was to examine the influence of CTH on the prediction of outcome and to study the effect of different types of SVD in a patient cohort with bilateral high grade ICAS.

## Methods

The study was approved by the ethics committee of the Ruprecht-Karls-University Heidelberg (statement S-330/2012). Due to the retrospective nature of this study, informed written consent was waived and patient data did not have to be anonymized or de-identified.

### Patient selection

Consecutive patients with bilateral high-grade (≥70% according to the North American Symptomatic Carotid Endarterectomy Trial Collaborators [19]) ICAS or internal carotid artery occlusion who underwent PWI between 2009 and 2014 were retrospectively selected from the hospital database. Degree of stenosis was measured using Doppler and Duplex sonography at our tertiary care hospital. Age, gender, degree of stenosis, presence of acute clinical symptoms (symptomatic vs. asymptomatic stenosis), symptomatic hemisphere, risk factors, National Institute of Health Stroke Scale (NIHSS) score on admission and modified Rankin scale (mRS) score on admission and at discharge were recorded. mRS was scored by an experienced vascular neurologist who was blinded to this analysis but not to the clinical course. Favorable outcome was defined as a mRS from 0–2, showing the ability to live independently. This definition is frequently used in stroke trials [20]. One patient with a premorbid mRS of 3 was included. For this patient an unchanged mRS was defined as favorable outcome as well.

### Image Acquisition

Images were acquired during routine clinical diagnostics using a 3 Tesla MR system (Magnetom Tim Trio or Verio with identical technical parameters, Siemens Healthcare, Erlangen, Germany) with a 12-channel head-matrix coil. For dynamic susceptibility contrast perfusion imaging, 0.1 mmol/kg gadolinium based contrast medium (Dotarem^®^, Guerbet) was administered and images were obtained with a T2-weighted gradient recalled echo (GRE) echo planar imaging (EPI) sequence (TE 35 ms, TR 1920 ms, FoV 240 x 240 mm, slice thickness 5 mm, 75 dynamic scans, with injection of 0.1 mmol/kg Dotarem^®^ 3.5 ml/s using a power injector after the third frame). The selected acquisition parameters resulted in an acquisition time of 2:31 for the PWI sequence.

### Image Analysis

T_max_ maps and the corresponding automatic and manual AIF were calculated using sSVD, cSVD and oSVD with the Olea-Sphere^®^ software (Olea Medical^®^, La Ciotat, France). Whole brain automatic detection for the arterial input function [21] and block-circulant matrix without minimization of oscillation single value decomposition deconvolution (cSVD, truncation threshold 0.1) were used. No model fitting for smoothing was applied. Motion correction was achieved using an algorithm with pairwise in-plane rigid co-registration of all raw images of a given slice with a well-chosen reference image over time. It is based on minimizing a robust and computationally friendly distance between this reference image and the target image. In order to avoid local minima, a quick, coarse grain registration algorithm based on geometric information is used to initialize the fine grain minimization algorithm. CTH maps were created using the Perfusion Graphical User Interface (PGUI, http://www.cfin.au.dk/software/penguin). The AIFs were selected based on an algorithm similar to the one used in Olea-Sphere^®^ and showed no relevant differences. Hypoperfusion was quantified in a volumetric approach. The maps were grouped by values and the respective volumes were computed using in-house developed software created with MATLAB (MathWorks^®^, Natick, MA, USA). For T_max_ the groups were: ≥ 4s, ≥ 6s, ≥ 8s and ≥ 10s and for CTH: ≥ 5s, ≥ 10s, ≥ 15s, ≥ 20s, ≥ 25s, ≥ 30s. All images were manually checked and corrected for artifacts using ITK-SNAP [22]. PWI images were co-registered with T2-images to facilitate artifact detection using a Statistical Parametric Mapping (SPM) based algorithm. Image reading was done blinded to outcome parameters.

### Statistical Analysis

Statistical analysis was performed with Microsoft Excel^®^ Version 2010 and IBM SPSS^®^ Version 21. Pretesting for normal distribution was not performed to avoid error accumulation [23]. Hence, paired group analysis was performed with the Friedman test and univariate analysis was performed using Mann-Whitney-U test. ROC-curve analysis was run including thresholds in case of positive classification and assuming a non-parametric distribution of the area under the curve. To facilitate comparison of the parameters, the same sensitivity value for all parameters was chosen to be reported with the corresponding specificity value in the results section. An α-Level of 0.05 was chosen. Two-sided p-values are reported throughout. P-values of the post-hoc analysis of the Friedman-test are adjusted p-values.

## Results

18 patients with bilateral high grade symptomatic or asymptomatic ICAS who were admitted to our hospital between 2009 and 2014 were included in the study. Baseline data of the patients are summarized in Table 1. 15 patients were admitted because of TIA or stroke, 2 because of syncopes and 1 for elective stenting of ICAS (S1 Table). The study group included mainly symptomatic patients with mild and moderate ischemic strokes (median NIHSS score of 3). 8 patients (44%) underwent carotid artery stenting or carotid endarterectomy and only one patient suffered from a periprocedural complication (aneurysm of the femoral artery after carotid artery stenting requiring surgical treatment).

**Table 1:** Baseline characteristics. Data is presented as median (interquartile range) or number (percentage).

N	18
Age	71 (58; 76)
Male Gender	9 (50%)
Degree of stenosis	
Left	90 (80; 100)
Right	90 (70; 100)
Symptomatic	15 (83%)
Left sided symptomatic hemisphere	6 (33%)
Risk factors:	
Atrial fibrillation	1 (6%)
Peripheral artery disease	4 (22%)
Coronary heart disease	4 (22%)
Current Smoker	7 (39%)
Hypercholesterolemia	8 (44%)
Hypertension	12 (67%)
Diabetes mellitus	5 (28%)
NIHSS score on admission	3 (2; 12)

NIHSS: National Institute of Health Stroke Scale

### Deconvolution analysis

The choice of deconvolution technique affected the definition of tissue displaying abnormal T_max_. The volume of tissue defined as having T_max_ ≥ 10s was significantly lower using sSVD compared to oSVD (median difference 4.33 ml [1.78;29.43] adjusted p<0.001). This was also true for the volume of tissue derived from sSVD compared to that from cSVD (median difference 5.07 ml [IQR 0.91;12.41]; adjusted p = 0.011). For T_max_ ≥ 8s, the volume was again lower using sSVD compared to cSVD (median difference 0.37 ml [0;1.24] adjusted p = 0.030). Maps generated with the delay-insensitive oSVD method were used in subsequent calculations to exclude bias caused by tracer arrival delays.

### Outcome analysis using T_max_ and CTH

**Fig 1:**
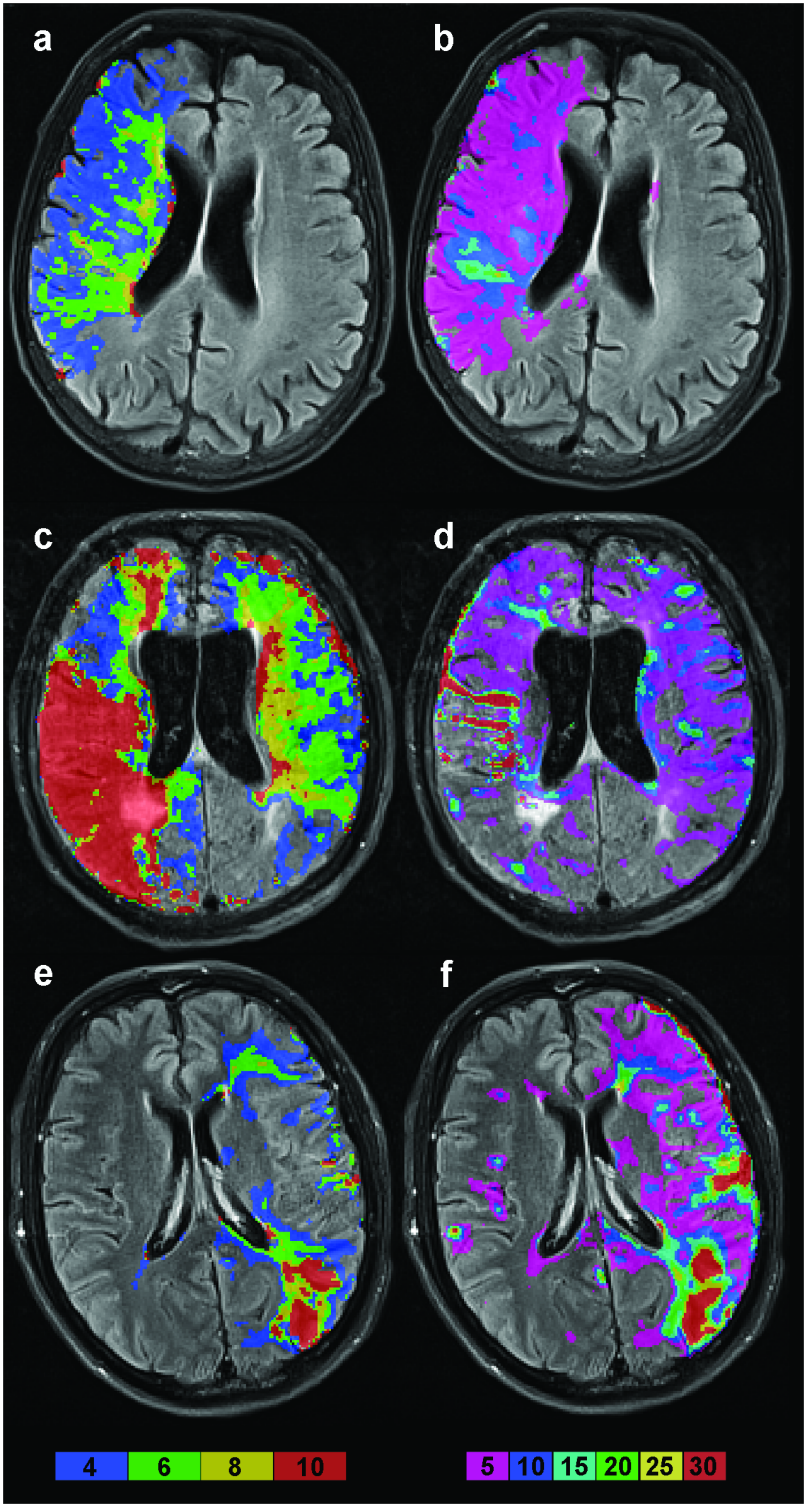
Tmax and CTH maps for three patients. Each row shows the most representative images of one patient. The first column depicts Tmax, the second CTH maps. Color-coded bars show Tmax and CTH values in seconds, respectively. The first patient (a, b) had perfusion abnormalities in the same region for Tmax and CTH with differing parts of that region being most severely affected. The second patient (c, d) shows a severe Tmax restriction while only slightly elevated CTH values can be seen. In the third patient (e, f) the profile for both Tmax and CTH seems to be comparable in the anterior and posterior middle cerebral artery border zone, while it is different in the temporoparietal region.

T_max_ maps were found to be markedly different from CTH maps (Fig 1). In the univariate analysis, only the volume of T_max_≥4s was significantly lower in the patients with favorable outcome compared to those with unfavorable outcome. We then analyzed CTH maps that were clustered in values of ≥5s, ≥10s, ≥15s, ≥20s, ≥25s and ≥30s. CTH maps could be generated for all patients. We assessed the total volume of tissue with elevated CTH values, the volume in each hemisphere, in the symptomatic and in the asymptomatic hemisphere (S2 Table). The volume of tissue with elevated CTH was significantly lower in patients with favorable compared to patients with unfavorable outcome for the following parameters: Left sided volume with CTH ≥ 5s; left sided volume with CTH ≥ 10s; left sided and total volume with CTH ≥ 15s; left sided and total volume with CTH ≥ 20s; left sided and total volume with CTH ≥ 25s; left sided and total volume with CTH ≥ 30s (Table 2).

**Table 2:** Parameters of ROC-curve (AUC, standard error, CI) and univariate analysis (in median and [IQR]) for all perfusion parameters with significant differences.

Perfusion parameter	AUC	standard error	CI		Volume of hypoperfusion		p-value
					Favorable outcome (n = 11)	Unfavorable outcome (n = 7)	
Tmax4oSVD total	0.803	0.111	0.585	1.000	18.28 ml [10.05;97.76]	114.03 ml [39.96;159.96]	0.048
CTH 5s left	0.773	0.122	0.534	1.000	43.94 ml [5.58;86.84]	130.01 ml [50.30;144.58]	0.035
CTH 10s left	0.879	0.094	0.694	1.000	10.32 ml [0.98;13.17	31.68 ml [16.13;40.84]	0.004
CTH 15s total	0.803	0.107	0.592	1.000	2.76 ml [0.99;16.79]	19.56 ml [8.15;23.51]	0.027
CTH 15s left	0.924	0.068	0.791	1.000	1.67 ml [0.39;3.64]	9.01 ml [6.23;16.26]	0.001
CTH 20s total	0.788	0.111	0.571	1.000	0.82 ml [0.45;5.81]	8.82 ml [2.15;9.73]	0.044
CTH 20s left	0.909	0.073	0.766	1.000	0.50 ml [0.18;1.51]	3.16 ml [1.95;8.07]	0.002
CTH 25s total	0.788	0.111	0.571	1.000	0.39 ml [0.20;2.21]	3.90 ml [1.36;5.27]	0.044
CTH 25s left	0.894	0.078	0.742	1.000	0.22 ml [0.07;0.63]	1.33 ml [0.74;5.03]	0.003
CTH 30s total	0.833	0.101	0.635	1.000	0.62 ml [0.12;3.02]	4.33 ml [2.80;11.07]	0.015
CTH 30s left	0.773	0.120	0.537	1.000	0.35 ml [0.00;1.22]	2.39 ml [0.65;10.88]	0.044

ROC-curve analysis revealed that the total volume with CTH ≥ 30s with a threshold of 2.69 ml yielded the best result when both hemispheres were taken into account (sensitivity 83%, specificity 73%, area under the curve [AUC] 0.833 [CI 0.635; 1.000]; p = 0.027). In comparison, the volume of T_max_ ≥ 4s with a threshold of 36.69 ml led to a sensitivity of 83%, but a specificity of only 64% (AUC 0.803 [CI 0.585;1.000]; p = 0.044). Interestingly, the highest AUC was found for left sided volume with CTH ≥ 15s (sensitivity 83%, specificity 91% with a threshold of 5.31 ml, AUC 0.924 [CI 0.791;1.000]; p = 0.005; Table 2). To exclude that CTH and T_max_ are comparable measurements of the same effect, we calculated CTH in the regions of T_max_ ≥4s, ≥6s, ≥8s and ≥10s, and found no significant difference of the CTH values between the regions (p = 0.058). In addition, the influence of the parametric calculation on the superior diagnostic accuracy of CTH was studied by generating parametric T_max_ maps. Although the volumes for all thresholds were significantly lower from T_max_ values calculated with sSVD, cSVD and oSVD, they did not differentiate between patients with favorable and unfavorable outcome in univariate analysis (p = 0.180/0.180/0.216/0.350 for total volume of parametric Tmax ≥ 4s/6s/8s/10s, respectively).

## Discussion

In the current study, we found CTH to be a novel imaging marker that is superior to T_max_ in the prediction of short term outcome in patients with bilateral high grade ICAS. The difference in the predictive capacity could reflect the importance of microvascular changes in these patients. While T_max_ characterizes macrovascular perfusion, CTH correlates to capillary flow patterns [12]. It is plausible that microemboli which are often caused by ICAS could disturb microvascular blood flow which has already been shown in an animal model [24]. This is more likely in regions with severe hypoperfusion that are commonly found in bilateral high grade ICAS due to a restricted washout of emboli [25] which in turn could lead to infarct progression and a worse short term outcome. In addition, microvascular changes can be found in any patient with cerebrovascular risk factors as hypertension, diabetes or smoking which are common risk factors in our population and can lead to cognitive decline [15,26,27]. Therefore, CTH maps might be an additional diagnostic tool to estimate the potential benefit of a recanalization procedure in patients with ICAS.

The superiority of CTH compared to T_max_ might be related to its computation. T_max_ is calculated as the time to the maximum of the residue function, making it susceptible to delay and dispersion of the intravascular tracer concentration time curve between its site of measurement and the tissue voxel [6]. Delay and dispersion frequently occur in bilateral high grade ICAS and could lead to distortions in the T_max_ values of these patients. The estimation of CTH is based on a flexible model of microvascular transport and offers delay insensitivity as well as more favorable noise progression properties than standard SVD techniques [11]. More importantly, the T_max_ parameter does not specifically differentiate between large vessel flow phenomena and microvascular flow heterogeneity.

This study is the first to examine the CTH parameter as a predictor in ICAS, and we therefore used several thresholds in our volumetric approach. The predictive effect was evident across a range of thresholds. Although 15s seems to be the best threshold in our patient cohort this has to be tested and verified in other patients with ischemic stroke. Another rather surprising finding was that higher left sided volume of increased CTH has an even higher diagnostic accuracy. Recently, a study described that atherosclerotic plaque in the left carotid artery is more vulnerable than in the right [28]. According to the authors this could explain why infarction is more common in the left hemisphere in other studies [29,30]. We postulate that these frequent emboli could cause a higher rate of infarction in hypoperfused tissue due to a decreased washout of emboli which has been shown to be a synergetic link between embolic and hemodynamic infarctions in patients with carotid artery occlusion [25]. At least in our population, left sided hypoperfusion seemed to be more predictive for outcome than the hypoperfusion on the symptomatic side.

Our results also revealed differences in the volumetric measurements depending on the type of deconvolution. This contradicts previous findings that studied T_max_ with simulations of delays between -4 and +4 seconds in 0.5–second increments [6]. Several factors might account for this difference. Firstly, the simulations were calculated with an optimal AIF, subjected to dispersion as modeled by an exponential vascular transport function. These conditions may not capture the vascular delay and dispersion in patients with bilateral high grade ICAS. Secondly, although the values of T_max_ are similar between sSVD and oSVD in the publication by Calamante et al. they did find that the rate of increase in T_max_ as a function of delay is higher in sSVD compared to oSVD. This could explain why the difference in our patients only occurred for higher T_max_ thresholds.

We hypothesize that CTH changes also have an effect on clinical outcome in patients with asymptomatic carotid artery stenosis as two of them had recurrent syncopes which could have been the consequence of poor collateral flow from the anterior circulation. Moreover, relevant hypoperfusion is associated with cognitive impairment that could have caused a worse clinical outcome independent of ischemic lesions [31].

Although susceptibility weighted imaging has been shown to predict clinically relevant hypoperfusion in ICAS [32], we did not use it in the current study as it requires the detection of an asymmetrical cortical vessel sign which would most likely not be found in bilateral high grade ICAS.

Main limitation of the current study is the retrospective design as well as the small number of included patients. This small patient number impeded an adjustment to known predictors of outcome and treatment modalities (e.g. stenting or carotid endarterectomy) after stroke. Especially the treatment modality exerts a strong influence on outcome in patients with high grade carotid artery stenosis which might have led to a confounding bias. In addition, using the dichotomized mRS as endpoint has its limitations. The dichotomization mainly discriminates the ability to live independently which might not be suitable to judge short term outcome. This endpoint also might have led to an oversimplification which can in turn lead to a distorted interpretation of the results. Moreover, short term outcome is quite dynamic and might have changed considerably at a later time point. Unfortunately, data at three months after discharge were not available. Inaccuracy of the degree of stenosis may have occurred as it was measured using Doppler and Duplex sonography. Furthermore, it would have been useful to study early follow-up images to test for the hypothesis of infarct growth in our patients. Future studies should assess the risk of recurrent stroke or the clinical outcome after several months in a prospective design.

## Conclusion

In the current study we found CTH to be a predictor of short term outcome in patients with bilateral high grade ICAS, which is superior to T_max_. Due to the severe perfusion abnormalities in this patient group, and especially to limit delay artifacts, oSVD, cSVD, or model-based parametric deconvolution should be preferred over sSVD and similar delay sensitive techniques. Larger, prospective trials should aim at confirming these effects and evaluating the thresholds for CTH in other stroke patients.

## Supporting Information

S1 Table Additional baseline characteristics for all patients. (DOCX)

S2 Table Volumes (in ml) of altered perfusion for different parameters in patients with favorable and unfavorable outcome. (DOCX)

## Abbreviations

AIF: Arterial input function
AUC: Area under the curve
CI: Confidence interval
cSVD: Block-circulant singular value decomposition
CTH: Capillary transit time heterogeneity
ICAS: internal carotid artery stenosis
IQR: interquartile range
mRS: modified Rankin Scale
NIHSS: National Institute of Health Stroke Scale Score
oSVD: Oscillation-index cSVD
PWI: Perfusion weighted imaging
ROC: Receiver operating characteristic
sSVD: Standard truncated singular value decomposition
T_max_: Time to the maximum of the residue curve

## References

[1] Ostergaard L, Sorensen AG, Kwong KK, Weisskoff RM, Gyldensted C, Rosen BR (1996) High resolution measurement of cerebral blood flow using intravascular tracer bolus passages. Part II: Experimental comparison and preliminary results. Magn Reson Med 36: 726–736. 8916023 10.1002/mrm.1910360511

[2] Sorensen AG, Copen WA, Ostergaard L, Buonanno FS, Gonzalez RG, Rordorf G, et al (1999) Hyperacute stroke: simultaneous measurement of relative cerebral blood volume, relative cerebral blood flow, and mean tissue transit time. Radiology 210: 519–527. 10207439 10.1148/radiology.210.2.r99fe06519

[3] Davis SM, Donnan GA, Parsons MW, Levi C, Butcher KS, Peeters A, et al (2008) Effects of alteplase beyond 3 h after stroke in the Echoplanar Imaging Thrombolytic Evaluation Trial (EPITHET): a placebo-controlled randomised trial. Lancet Neurol 7: 299–309. 10.1016/S1474-4422(08)70044-9 18296121

[4] Albers GW, Thijs VN, Wechsler L, Kemp S, Schlaug G, Skalabrin E, et al (2006) Magnetic resonance imaging profiles predict clinical response to early reperfusion: the diffusion and perfusion imaging evaluation for understanding stroke evolution (DEFUSE) study. Ann Neurol 60: 508–517. 17066483 10.1002/ana.20976

[5] Lansberg MG, Straka M, Kemp S, Mlynash M, Wechsler LR, Jovin TG, et al (2012) MRI profile and response to endovascular reperfusion after stroke (DEFUSE 2): a prospective cohort study. The Lancet Neurology 11: 860–867. 10.1016/S1474-4422(12)70203-X 22954705 PMC4074206

[6] Calamante F, Christensen S, Desmond PM, Ostergaard L, Davis SM, Connelly A (2010) The physiological significance of the time-to-maximum (Tmax) parameter in perfusion MRI. Stroke 41: 1169–1174. 10.1161/STROKEAHA.110.580670 20413735

[7] Shih LC, Saver JL, Alger JR, Starkman S, Leary MC, Vinuela F, et al (2003) Perfusion-weighted magnetic resonance imaging thresholds identifying core, irreversibly infarcted tissue. Stroke 34: 1425–1430. 12738899 10.1161/01.STR.0000072998.70087.E9

[8] Ostergaard L, Weisskoff RM, Chesler DA, Gyldensted C, Rosen BR (1996) High resolution measurement of cerebral blood flow using intravascular tracer bolus passages. Part I: Mathematical approach and statistical analysis. Magn Reson Med 36: 715–725. 8916022 10.1002/mrm.1910360510

[9] Wu O, Ostergaard L, Weisskoff RM, Benner T, Rosen BR, Sorensen AG (2003) Tracer arrival timing-insensitive technique for estimating flow in MR perfusion-weighted imaging using singular value decomposition with a block-circulant deconvolution matrix. Magn Reson Med 50: 164–174. 12815691 10.1002/mrm.10522

[10] Mouridsen K, Friston K, Hjort N, Gyldensted L, Ostergaard L, Kiebel S (2006) Bayesian estimation of cerebral perfusion using a physiological model of microvasculature. Neuroimage 33: 570–579. 16971140 10.1016/j.neuroimage.2006.06.015

[11] Mouridsen K, Hansen MB, Ostergaard L, Jespersen SN (2014) Reliable estimation of capillary transit time distributions using DSC-MRI. J Cereb Blood Flow Metab 34: 1511–1521. 10.1038/jcbfm.2014.111 24938401 PMC4158667

[12] Jespersen SN, Ostergaard L (2012) The roles of cerebral blood flow, capillary transit time heterogeneity, and oxygen tension in brain oxygenation and metabolism. J Cereb Blood Flow Metab 32: 264–277. 10.1038/jcbfm.2011.153 22044867 PMC3272609

[13] Østergaard L, Sorensen AG, Chesler DA, Weisskoff RM, Koroshetz WJ, Wu O, et al (2000) Combined diffusion-weighted and perfusion-weighted flow heterogeneity magnetic resonance imaging in acute stroke. Stroke 31: 1097–1103. 10797171 10.1161/01.str.31.5.1097

[14] Ostergaard L, Jespersen SN, Mouridsen K, Mikkelsen IK, Jonsdottir KY, Tietze A, et al (2013) The role of the cerebral capillaries in acute ischemic stroke: the extended penumbra model. J Cereb Blood Flow Metab 33: 635–648. 10.1038/jcbfm.2013.18 23443173 PMC3652700

[15] Ostergaard L, Jespersen SN, Engedahl T, Gutierrez Jimenez E, Ashkanian M, Hansen MB, et al (2015) Capillary dysfunction: its detection and causative role in dementias and stroke. Curr Neurol Neurosci Rep 15: 37 10.1007/s11910-015-0557-x 25956993 PMC4441906

[16] Neumann-Haefelin T, Wittsack HJ, Fink GR, Wenserski F, Li TQ, Seitz RJ, et al (2000) Diffusion- and perfusion-weighted MRI: influence of severe carotid artery stenosis on the DWI/PWI mismatch in acute stroke. Stroke 31: 1311–1317. 10835450 10.1161/01.str.31.6.1311

[17] Mundiyanapurath S, Ringleb PA, Diatschuk S, Eidel O, Burth S, Floca R, et al (2016) Time-dependent parameter of perfusion imaging as independent predictor of clinical outcome in symptomatic carotid artery stenosis. BMC Neurol 16: 50 10.1186/s12883-016-0576-5 27094741 PMC4837540

[18] Reinhard M, Müller T, Roth M, Guschlbauer B, Timmer J, Hetzel A (2003) Bilateral severe carotid artery stenosis or occlusion–cerebral autoregulation dynamics and collateral flow patterns. Acta neurochirurgica 145: 1053–1060. 14663562 10.1007/s00701-003-0137-8

[19] North-American-Symptomatic-Carotid-Endarterectomy-Trial-Collaborators (1991) Beneficial effect of carotid endarterectomy in symptomatic patients with high-grade carotid stenosis. N Engl J Med 325: 445–453. 1852179 10.1056/NEJM199108153250701

[20] Broderick JP, Palesch YY, Demchuk AM, Yeatts SD, Khatri P, Hill MD, et al (2013) Endovascular therapy after intravenous t-PA versus t-PA alone for stroke. N Engl J Med 368: 893–903. 10.1056/NEJMoa1214300 23390923 PMC3651875

[21] Mouridsen K, Christensen S, Gyldensted L, Ostergaard L (2006) Automatic selection of arterial input function using cluster analysis. Magn Reson Med 55: 524–531. 16453314 10.1002/mrm.20759

[22] Yushkevich PA, Piven J, Hazlett HC, Smith RG, Ho S, Gee JC, et al (2006) User-guided 3D active contour segmentation of anatomical structures: significantly improved efficiency and reliability. Neuroimage 31: 1116–1128. 16545965 10.1016/j.neuroimage.2006.01.015

[23] Rochon J G M, Kieser M (2012) To test or not to test: Preliminary assessment of normality when comparing two independent samples. BMC Med Res Methodol 12. 10.1186/1471-2288-12-81 PMC3444333 22712852

[24] Srinivasan VJ, Yu E, Radhakrishnan H, Can A, Climov M, Leahy C, et al (2015) Micro-heterogeneity of flow in a mouse model of chronic cerebral hypoperfusion revealed by longitudinal Doppler optical coherence tomography and angiography. J Cereb Blood Flow Metab 35: 1552–1560. 10.1038/jcbfm.2015.175 26243708 PMC4640323

[25] Caplan LR, Hennerici M (1998) Impaired clearance of emboli (washout) is an important link between hypoperfusion, embolism, and ischemic stroke. Arch Neurol 55: 1475–1482. 9823834 10.1001/archneur.55.11.1475

[26] Iadecola C (2013) The pathobiology of vascular dementia. Neuron 80: 844–866. 10.1016/j.neuron.2013.10.008 24267647 PMC3842016

[27] Prince M, Patel V, Saxena S, Maj M, Maselko J, Phillips MR, et al (2007) No health without mental health. Lancet 370: 859–877. 17804063 10.1016/S0140-6736(07)61238-0

[28] Selwaness M, van den Bouwhuijsen Q, van Onkelen RS, Hofman A, Franco OH, van der Lugt A, et al (2014) Atherosclerotic plaque in the left carotid artery is more vulnerable than in the right. Stroke 45: 3226–3230. 10.1161/STROKEAHA.114.005202 25228259

[29] Hedna VS, Bodhit AN, Ansari S, Falchook AD, Stead L, Heilman KM, et al (2013) Hemispheric differences in ischemic stroke: is left-hemisphere stroke more common? J Clin Neurol 9: 97–102. 10.3988/jcn.2013.9.2.97 23626647 PMC3633197

[30] Foerch C, Misselwitz B, Sitzer M, Berger K, Steinmetz H, Neumann-Haefelin T (2005) Difference in recognition of right and left hemispheric stroke. Lancet 366: 392–393. 16054939 10.1016/S0140-6736(05)67024-9

[31] Wirth M, Pichet Binette A, Brunecker P, Kobe T, Witte AV, Floel A (2016) Divergent regional patterns of cerebral hypoperfusion and gray matter atrophy in mild cognitive impairment patients. J Cereb Blood Flow Metab. 10.1177/0271678X16641128 PMC5363461 27037094

[32] Mundiyanapurath S, Ringleb PA, Diatschuk S, Burth S, Möhlenbruch M, Floca RO, et al (2016) Cortical vessel sign on susceptibility weighted imaging reveals clinically relevant hypoperfusion in internal carotid artery stenosis. European Journal of Radiology 85: 534–539. 10.1016/j.ejrad.2015.12.020 26860664

