# Supplementary material for: Capillary Transit Time Heterogeneity Is Associated with Modified Rankin Scale Score at Discharge in Patients with Bilateral High Grade Internal Carotid Artery Stenosis
Source: PLoS One. 2016 Jun 23;11(6):e0158148. doi: 10.1371/journal.pone.0158148 (PMC4919050; doi:10.1371/journal.pone.0158148)
Supplement: S1 Table — (DOCX) [file pone.0158148.s001.docx]

**S1 Table: Detailed baseline characteristics on included patients**

| Patient | Symptomatic stenosis | Presenting event | TOAST | NIHSS | Stroke territory | mRS on admission | mRS at discharge | Degree of stenosis left/right (NASCET) | Degree of stenosis left/right (ECST) |
| --- | --- | --- | --- | --- | --- | --- | --- | --- | --- |
| 1 | yes | multiple TIA | atherosclerosis | 0 | - | 0 | 0 | 100/70 | 100/80 |
| 2 | no | stenting of stenosis | - | 0 | - | 0 | 1 | 70/100 | 80/100 |
| 3 | yes | minor stroke | atherosclerosis | 2 | PACS | 3 | 3 | 90/70 | 95/80 |
| 4 | no | recurrent syncopes | - | 0 | - | 1 | 1 | 80/70 | 90/80 |
| 5 | yes | major stroke | atherosclerosis | 6 | PACS | 2 | 4 | 90/80 | 95/90 |
| 6 | yes | major stroke | atherosclerosis | 3 | PACS | 0 | 2 | 80/100 | 90/100 |
| 7 | yes | major stroke | atherosclerosis | 19 | PACS | 2 | 5 | 100/100 | 100/100 |
| 8 | yes | major stroke | atherosclerosis | 13 | PACS | 1 | 3 | 80/90 | 90/95 |
| 9 | yes | major stroke | atherosclerosis | 2 | PACS | 0 | 0 | 90/100 | 95/100 |
| 10 | yes | major stroke | atherosclerosis | 17 | PACS | 1 | 3 | 80/100 | 90/100 |
| 11 | yes | single TIA | atherosclerosis | 1 | - | 1 | 1 | 100/70 | 100/80 |
| 12 | yes | major stroke | atherosclerosis | 3 | PACS | 0 | 0 | 90/100 | 95/100 |
| 13 | no | syncope | - | 0 | - | 0 | 2 | 80/90 | 90/95 |
| 14 | yes | major stroke | atherosclerosis | 3 | PACS | 0 | 2 | 70/90 | 80/95 |
| 15 | yes | major stroke | atherosclerosis | 9 | PACS | 0 | 2 | 90/100 | 95/100 |
| 16 | yes | major stroke | atherosclerosis | 6 | PACS | 2 | 3 | 100/70 | 100/80 |
| 17 | yes | major stroke | atherosclerosis | 10 | PACS | 2 | 6 | 90/100 | 95/100 |
| 18 | yes | major stroke | atherosclerosis | 25 | TACS | 0 | 5 | 100/80 | 100/90 |
