# Supplementary material for: Capillary Transit Time Heterogeneity Is Associated with Modified Rankin Scale Score at Discharge in Patients with Bilateral High Grade Internal Carotid Artery Stenosis
Source: PLoS One. 2016 Jun 23;11(6):e0158148. doi: 10.1371/journal.pone.0158148 (PMC4919050; doi:10.1371/journal.pone.0158148)
Supplement: S2 Table — (DOCX) [file pone.0158148.s002.docx]

**S2 Table: Volumes (in ml) of altered perfusion for different parameters in patients with favorable and unfavorable outcome**

|  | Unfavorable Outcome (n=7) | | | Favorable Outcome (n=11) | | | p-value |
| --- | --- | --- | --- | --- | --- | --- | --- |
|  | Median | 25% | 75% | Median | 25% | 75% |  |
| Tmax ≥ 4s |  |  |  |  |  |  |  |
| - Total | 114.03 | 39.96 | 160.00 | 18.28 | 10.05 | 97.76 | 0.048 |
| - Left | 22.29 | 11.71 | 105.64 | 6.76 | 1.52 | 26.82 | 0.149 |
| - Right | 57.13 | 4.26 | 100.48 | 9.44 | 4.08 | 49.30 | 0.301 |
| - Symptomatic | 83.64 | 27.91 | 121.18 | 23.65 | 7.72 | 88.60 | 0.181 |
| - Asymptomatic | 9.53 | 3.69 | 46.28 | 7.62 | 2.16 | 42.49 | 0.852 |
| Tmax ≥ 6s |  |  |  |  |  |  |  |
| - Total | 44.00 | 6.59 | 74.28 | 4.54 | 1.96 | 44.61 | 0.098 |
| - Left | 4.22 | 1.73 | 47.53 | 2.49 | 0.50 | 10.56 | 0.350 |
| - Right | 24.51 | 1.96 | 47.44 | 2.04 | 0.92 | 16.16 | 0.149 |
| - Symptomatic | 38.75 | 5.31 | 47.44 | 6.55 | 1.63 | 40.78 | 0.181 |
| - Asymptomatic | 2.07 | 1.28 | 25.90 | 2.46 | 0.89 | 14.52 | 1.000 |
| Tmax ≥ 8s |  |  |  |  |  |  |  |
| - Total | 12.00 | 2.74 | 36.29 | 2.66 | 0.81 | 15.12 | 0.216 |
| - Left | 2.84 | 1.17 | 16.58 | 1.68 | 0.37 | 5.86 | 0.462 |
| - Right | 6.38 | 1.42 | 22.18 | 1.37 | 0.49 | 7.97 | 0.149 |
| - Symptomatic | 10.45 | 1.83 | 22.18 | 4.42 | 1.13 | 17.52 | 0.414 |
| - Asymptomatic | 1.56 | 0.91 | 12.15 | 1.99 | 0.64 | 5.72 | 1.000 |
| Tmax ≥ 10s |  |  |  |  |  |  |  |
| - Total | 43.77 | 19.13 | 217.28 | 17.52 | 7.89 | 90.59 | 0.216 |
| - Left | 23.52 | 7.94 | 56.86 | 13.25 | 3.24 | 38.82 | 0.404 |
| - Right | 12.37 | 9.66 | 164.70 | 8.14 | 4.27 | 51.77 | 0.180 |
| - Symptomatic | 33.05 | 10.28 | 164.70 | 17.55 | 7.08 | 55.55 | 0.491 |
| - Asymptomatic | 11.71 | 7.37 | 43.52 | 12.22 | 4.68 | 49.04 | 0.852 |
| CTTH ≥ 5s |  |  |  |  |  |  |  |
| - Total | 171.18 | 93.85 | 255.66 | 63.32 | 19.38 | 144.70 | 0.085 |
| - Left | 130.01 | 50.30 | 144.58 | 43.94 | 5.58 | 86.84 | 0.035 |
| - Right | 51.76 | 26.60 | 91.26 | 19.38 | 12.88 | 77.54 | 0.328 |
| - Symptomatic | 70.39 | 51.76 | 130.01 | 58.74 | 14.50 | 131.92 | 0.694 |
| - Asymptomatic | 50.30 | 35.46 | 144.44 | 45.06 | 6.17 | 86.38 | 0.336 |
| CTTH ≥ 10s |  |  |  |  |  |  |  |
| - Total | 52.14 | 25.74 | 57.04 | 12.98 | 3.18 | 34.89 | 0.056 |
| - Left | 31.68 | 16.13 | 40.84 | 10.32 | 0.98 | 13.17 | 0.004 |
| - Right | 9.61 | 5.06 | 35.00 | 2.66 | 1.16 | 17.63 | 0.375 |
| - Symptomatic | 35.00 | 6.75 | 40.84 | 11.67 | 2.20 | 39.39 | 0.463 |
| - Asymptomatic | 16.20 | 6.37 | 31.68 | 11.85 | 1.18 | 16.52 | 0.232 |
| CTTH ≥ 15s |  |  |  |  |  |  |  |
| - Total | 19.56 | 8.15 | 23.51 | 2.76 | 0.99 | 16.79 | 0.027 |
| - Left | 9.01 | 6.23 | 16.26 | 1.67 | 0.39 | 3.64 | 0.001 |
| - Right | 3.35 | 1.44 | 14.49 | 1.11 | 0.40 | 9.36 | 0.246 |
| - Symptomatic | 14.49 | 1.44 | 17.84 | 2.65 | 0.62 | 14.68 | 0.336 |
| - Asymptomatic | 5.31 | 2.03 | 9.01 | 2.08 | 0.45 | 4.55 | 0.152 |
| CTTH ≥ 20s |  |  |  |  |  |  |  |
| - Total | 8.82 | 2.15 | 9.73 | 0.82 | 0.45 | 5.81 | 0.044 |
| - Left | 3.16 | 1.95 | 8.07 | 0.50 | 0.18 | 1.51 | 0.002 |
| - Right | 1.29 | 0.56 | 6.41 | 0.35 | 0.15 | 4.77 | 0.211 |
| - Symptomatic | 6.41 | 0.56 | 9.11 | 1.00 | 0.23 | 4.59 | 0.152 |
| - Asymptomatic | 1.95 | 1.29 | 2.40 | 0.77 | 0.18 | 1.69 | 0.094 |
| CTTH ≥ 25s |  |  |  |  |  |  |  |
| - Total | 3.90 | 1.36 | 5.27 | 0.39 | 0.20 | 2.21 | 0.044 |
| - Left | 1.33 | 0.74 | 5.03 | 0.22 | 0.07 | 0.63 | 0.003 |
| - Right | 0.79 | 0.24 | 3.98 | 0.17 | 0.08 | 1.73 | 0.179 |
| - Symptomatic | 2.80 | 0.32 | 5.03 | 0.61 | 0.11 | 2.49 | 0.152 |
| - Asymptomatic | 0.79 | 0.54 | 1.11 | 0.33 | 0.06 | 0.64 | 0.054 |
| CTTH ≥ 30s |  |  |  |  |  |  |  |
| - Total | 4.33 | 2.80 | 11.07 | 0.62 | 0.12 | 3.02 | 0.015 |
| - Left | 2.39 | 0.65 | 10.88 | 0.35 | 0.00 | 1.22 | 0.044 |
| - Right | 1.64 | 0.19 | 10.67 | 0.26 | 0.09 | 1.68 | 0.126 |
| - Symptomatic | 2.39 | 0.28 | 10.88 | 0.97 | 0.10 | 3.28 | 0.152 |
| - Asymptomatic | 1.64 | 0.31 | 4.05 | 0.26 | 0.00 | 0.91 | 0.165 |
